# Influence of underlying condition and performance of sepsis bundle in very old patients with sepsis: a nationwide cohort study

**DOI:** 10.1186/s13613-024-01415-x

**Published:** 2024-12-04

**Authors:** Duk Ki Kim, Soyun Kim, Da Hyun Kang, Hyekyeong Ju, Dong Kyu Oh, Su Yeon Lee, Mi Hyeon Park, Chae-Man Lim, YunKyong Hyon, Song I Lee, the Korean sepsis Alliance (KSA) investigators, Dong Kyu Oh, Chae-Man Lim, Kyeongman Jeon, Sunghoon Park, Yeon Joo Lee, Sang-Bum Hong, Gee Young Suh, Young-Jae Cho, Ryoung-Eun Ko, Sung Yoon Lim, Jeongwon Heo, Jae-myeong Lee, Kyung Chan Kim, Yeon Joo Lee, Youjin Chang, Kyeongman Jeon, Sang-Min Lee, Suk-Kyung Hong, Woo Hyun Cho, Sang Hyun Kwak, Heung Bum Lee, Jong-Joon Ahn, Gil Myeong Seong, Song I Lee, Sunghoon Park, Tai Sun Park, Su Hwan Lee, Eun Young Choi, Jae Young Moon

**Affiliations:** 1 Division of Allergy, Pulmonary, and Critical Care Medicine, Department of Internal Medicine, Chungnam National University Hospital, Chungnam National University School of Medicine, Munhwaro 282Jung Gu, Daejeon, 35015 Republic of Korea; 2 Department of Pulmonary and Critical Care Medicine, Dongkang Medical Center, Ulsan, Republic of Korea; 3 Department of Pulmonary and Critical Care Medicine, Asan Medical Center, Seoul, Republic of Korea; 4 Data-Analytic Research Team, National Institute for Mathematical Sciences, Daejon, Republic of Korea

**Keywords:** Sepsis, Septic shock, Surviving sepsis campaign, Very old patients

## Abstract

**Background:**

Sepsis is a life-threatening condition that affects individuals of all ages; however, it presents unique challenges in very old patients due to their complex medical histories and potentially compromised immune systems. This study aimed to investigate the influence of underlying conditions and the performance of sepsis bundle protocols in very old patients with sepsis.

**Methods:**

We conducted a nationwide cohort study of adult patients with sepsis prospectively collected from the Korean Sepsis Alliance Database. Underlying conditions, prognosis, and their association with sepsis bundle compliance in patients with sepsis aged ≥ 80 years were analyzed.

**Results:**

Among the 11,981 patients with sepsis, 3,733 (31.2%) were very old patients aged ≥ 80 years. In-hospital survivors (69.8%) were younger, less likely male, with higher BMI, lower Charlson Comorbidity Index, lower Clinical Frailty Scale, and lower Sequential Organ Failure Assessment (SOFA) scores. The in-hospital survivor group had lower lactate measurement but higher fluid therapy and vasopressor usage within the 1-h bundle. Similar trends were seen in the 3-h and 6-h bundles. Furthermore, in-hospital survivors were more likely to receive appropriate empiric antibiotics within 24 h. In-hospital mortality was associated with age, Clinical Frailty Scale, SOFA score, comorbidities, Life sustaining treatment issue, interventions in the ICU and vasopressor use in the 1-h sepsis bundle.

**Conclusions:**

Addressing underlying conditions and enhancing sepsis bundle adherence is crucial for better outcomes in very old patients with sepsis. Personalized approaches and increased awareness are essential. Further research should explore interventions to optimize sepsis care in this population.

**Supplementary Information:**

The online version contains supplementary material available at 10.1186/s13613-024-01415-x.

## Background

The world's population continues to age [1], and South Korea is one of the countries with a rapidly growing older population [2]. Aging is associated with a higher prevalence of chronic diseases [3] and increased hospital and intensive care unit (ICU) admissions [4, 5]. This demographic shift poses significant challenges and considerations for the healthcare system, particularly in the management of complex conditions such as sepsis in the very old population.

Sepsis, a critical condition characterized by a dysregulated host response to infection leading to life-threatening organ dysfunction, has been noted for its increasing severity [6], accounting for > 20% of ICU admissions in recent years [7]. Sepsis and its more severe form, septic shock, are associated with significantly higher mortality rates, particularly in older patients [8, 9]. Previous data has shown that age is a major predictor of mortality in patients with sepsis, which has contributed to a reluctance on the part of ICU physicians to admit older patients to the ICU [10], even if they meet clinical criteria for admission.

The Surviving Sepsis Campaign (SSC) guidelines were revised in 2018 and subsequently updated in 2021 to introduce an integrated 1-h bundle aimed at prompt initiation of resuscitation and management [11]. Despite the intention to improve outcomes, the reception of the 1-h bundle has been mixed, partly due to concerns about its staff shortages and implementation challenges [12]. In addition, the effectiveness of these revised guidelines in improving patient-centered outcomes has not been consistently observed [13–15].

The prognostic perspectives of very old patients with sepsis remain poorly understood, with outstanding questions regarding the impact of sepsis management strategies and patients' baseline health status on outcomes. This study aims to address this gap by investigating the relationship between the baseline health status of very old patients with sepsis (aged ≥ 80 years), sepsis management bundle performance, and patient prognosis, providing insights into individualized care strategies for this vulnerable population.

## Methods

Between September 2019 and December 2021, a prospective cohort study was conducted under the leadership of the Korean Sepsis Alliance, a national multicenter registry. The study involved 20 tertiary and university-affiliated hospitals across South Korea participating in sepsis management education programs. Adult patients aged ≥ 19 years diagnosed with sepsis or septic shock, according to the Third International Consensus Definitions for Sepsis and Septic Shock (Sepsis-3), were systematically identified and enrolled. The specific enrollment criteria for this study are outlined as follows; (1) Patients with community-onset sepsis (COS) who meet both of the following criteria: Adults aged 19 years or older who underwent blood culture testing in the emergency department and met at least two of the three qSOFA criteria upon presentation. (However, in cases where mental status assessment is challenging, patients must meet at least one of the two criteria; respiratory rate or systolic blood pressure). (2) Hospital-onset sepsis (HOS) patients aged 19 years or older, as identified by the rapid response teams of each hospital. The lead institution, Asan Medical Center in Seoul, conducted regular data quality audits to ensure the integrity of the data collected from these institutions. Ethics monitoring was provided by the institutional review boards of the participating hospitals, including specific approval from Chungnam National University Hospital (2019-11-048). Given the study's observational nature, which focused on standard care with minimal risk and no intervention, the requirement for informed consent was waived.

### Data recruitment and definition

Sepsis and septic shock are defined in the Sepsis-3 Guidelines. Sepsis is life-threatening organ dysfunction due to a dysregulated response to infection, defined as an increase in Sequential Organ Failure Assessment (SOFA) score of ≥ 2 points. Septic shock is defined as a severe state of sepsis characterized by persistent hypotension despite adequate fluid resuscitation, the need for vasopressors, and a lactate level of ≥ 2 mmol/L.

The study aimed to analyze the outcomes and treatment effects of sepsis in patients aged ≥ 80 years (the "very old" group). The methodology involved comprehensive follow-up of these patients from diagnosis until discharge or death. The enrollment target for each institution was determined based on the annual average number of sepsis patients at each hospital. Ultimately, a total of 11,981 patients were enrolled. Data were obtained through a systematic review of electronic medical records (EMR) from the participating hospitals. To minimize potential bias, data accuracy was ensured through a double-check process conducted by the principal investigators and research nurses at each institution. Additionally, data quality was centrally managed by the lead institution, Asan Medical Center, to maintain consistency and reliability across all study sites. Data were meticulously collected on multiple dimensions: demographic information such as age, sex, body mass index, comorbidities, pathogen type, site of infection, SOFA score assessing disease severity, the Clinical Frailty Scale, and the Charlson Comorbidity Index, Eastern Cooperative Oncology Group (ECOG) as an indication of the patient's underlying frailty. Additionally, details of the patient's care, including ICU admission, therapeutic interventions, and healthcare resources used, were collected. However, BMI data were missing for 583 patients, and hospital length of stay data were unavailable for 593 patients. In addition, in-hospital mortality was assessed to determine the patient's prognosis.

The primary outcome of this study was in-hospital mortality, while the secondary outcomes included hospital length of stay and the number of ICU stay days.

### Sepsis bundle performance

This study evaluates the implementation success of sepsis management bundles applied within the first 1, 3, and 6 h of patient admission, known as the 1-, 3-, and 6-h bundles, respectively. Implementation success of each bundle was determined based on the completion of all specified interventions within the specified timeframes. These interventions included the following: measurement of lactate levels, blood cultures, administration of broad-spectrum antibiotics, fluid therapy, and vasopressor use.

### Definition of bundle success:

One-hour bundle success is defined as the completion of all of the above interventions within the first hour of sepsis recognition.

Three-hour bundle success is defined as the completion of all interventions within three hours of sepsis recognition.

Six-hour bundle success is similarly defined, requiring completion of all interventions within six hours of sepsis recognition.

Appropriateness of initial empiric antibiotics (within 24 h) refers to the administration of appropriate empiric antibiotics within the first 24 h. The appropriateness of antimicrobial therapy was evaluated by the principal investigators (PIs) and research nurses at each participating hospital. For patients where antibiotic susceptibility was confirmed, the appropriateness was based on those results. In cases where susceptibility data were not available, the appropriateness was assessed according to the empiric antibiotic regimens recommended by established clinical guidelines.

### Statistical analysis

The statistical analysis of this study was performed using SPSS software version 25 (IBM Corp., Armonk, NY, USA). Categorical variables were presented as counts (n) and percentages (%) to provide a clear picture of the data distribution. Missing data were handled as Not Available (NA) and excluded from multivariable analysis. Continuous variables were presented as means and standard deviations to measure central tendency and dispersion within the data set quantitatively. We used t-tests to compare continuous variables between different groups robustly. For categorical variables, we used the chi-squared test or Fisher's exact test. The study also used a Cox regression model to identify factors associated with in-hospital mortality. For the variable selection in the Cox regression model, we included all significant variables identified through T-tests and Chi-square tests comparing in-hospital survivors and non-survival groups (p-value of < 0.10). These variables were first subjected to a univariate Cox regression analysis, and those that remained statistically significant were then included in the multivariable Cox regression analysis. The Cox regression model results were expressed as hazard ratio (HR) with 95% confidence interval (CI) to provide a comprehensive measure of the strength and direction of the association between the variable of interest and the mortality outcome. Statistical significance was set at a p-value of < 0.05.

## Results

### Characteristics of included patients

**Fig. 1:**
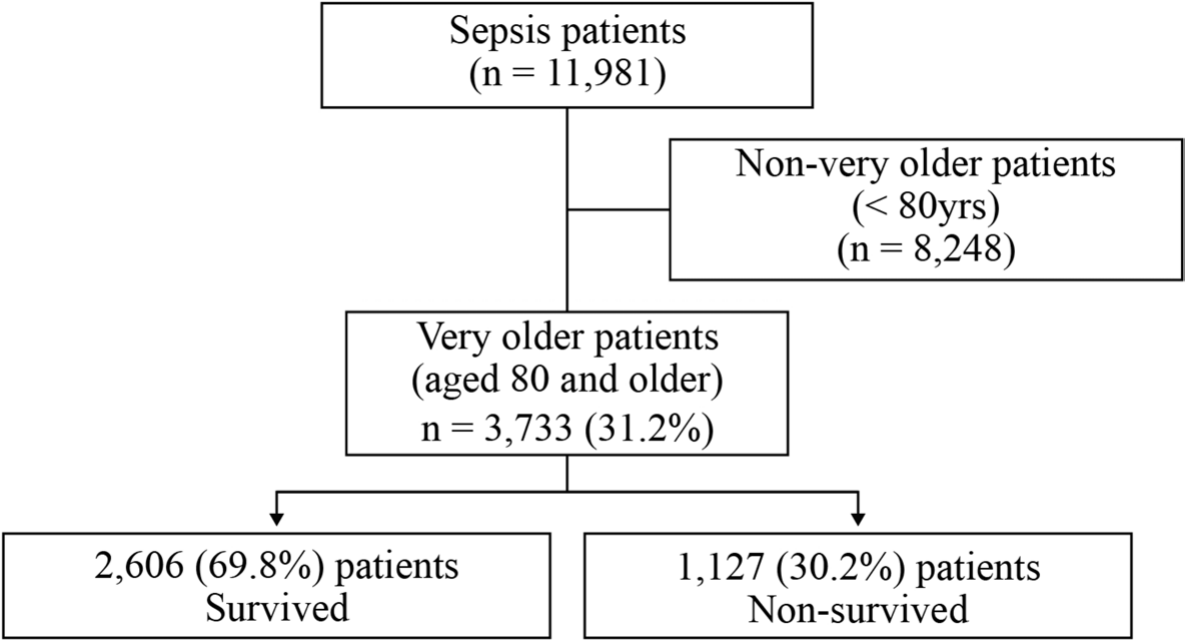
Flow chart of the enrolled patients

In the study, of the 11,981 patients diagnosed with sepsis, 3,733 (31.2%) were aged ≥ 80 years and classified as “very old” thus included in the analysis (Fig. 1). Of the very old patients, 2,606 (69.8%) survived (in-hospital survivor group), while 1,127 (30.2%) did not survive (non-survivor group). Comprehensive follow-up of all patients was conducted from the time of diagnosis until discharge or death.

Table 1 presents the patients’ baseline characteristics. Compared to the non-survivor group, the in-hospital survivor group had a slightly lower mean age (p = 0.041), a lower proportion of males (p = 0.001), and a higher mean body mass index (p < 0.001). The Charlson Comorbidity Index was significantly lower in the in-hospital survivor group compared to the non-survivor group (p < 0.001), reflecting fewer comorbidities. In addition, in-hospital survivors were less clinically frail and had better performance status compared to the non-survivor group (both p < 0.001). SOFA scores, a severity of organ failure indicator, were significantly higher in the non-survivor group than in the in-hospital survivor group (p < 0.001). The prevalence of solid tumors, hematologic malignancies, heart failure, and chronic obstructive pulmonary disease were differentially distributed across outcomes, with higher in the non-survivor group than in the in-hospital survivor group.

**Table 1:** Patient’s characteristics according to survivor

Characteristics	All patients	Survivor	Non-survivor	P-value
Patients (n)	3733	2606	1127	
Age, yr	85.2 ± 4.2	85.1 ± 4.2	85.4 ± 4.3	0.041
Male	1846 (49.5)	1241 (47.6)	605 (53.7)	0.001
Body mass index, kg/m^2^	21.14 ± 3.97	21.31 ± 4.01	20.76 ± 3.85	< 0.001
Resident of nursing care center	670 (17.9)	469 (18.0)	201 (17.8)	0.906
Charlson comorbidity index	6.5 ± 2.1	6.4 ± 2.0	6.8 ± 2.3	< 0.001
Clinical frailty scale	6.2 ± 1.7	6.1 ± 1.7	6.4 ± 1.6	< 0.001
ECOG	2.7 ± 1.2	2.7 ± 1.2	2.9 ± 1.1	< 0.001
SOFA score	6.3 ± 2.9	5.8 ± 2.5	7.6 ± 3.2	< 0.001
Underlying disease				
Diabetes Mellitus	1,285 (34.4)	905 (34.7)	380 (33.7)	0.551
Solid tumor	876 (23.5)	561 (21.5)	315 (28.0)	< 0.001
Hematologic malignancy	92 (2.5)	46 (1.8)	46 (4.1)	< 0.001
Chronic kidney disease	493 (13.2)	330 (12.7)	163 (14.5)	0.136
Liver disease	136 (3.6)	87 (3.3)	49 (4.3)	0.131
Heart failure	336 (9.0)	213 (8.2)	123 (10.9)	0.007
Chronic obstructive pulmonary disease	294 (7.9)	183 (7.0)	111 (9.8)	0.003
Cerebrovascular disease	970 (26.0)	711 (27.3)	259 (23.0)	0.006
Dementia	1,210 (32.4)	860 (33.0)	350 (31.1)	0.244

Data are presented as mean ± standard deviation or number (%), unless otherwise indicated

Table 2 presents the infection site and pathogen type. Pulmonary was the most common site of suspicion (52.2%), with a significant difference between the in-hospital survivor group (48.3%) and the non-survivor group (61.2%, p < 0.001). Compared to the non-survivor group, urinary tract infections were more common in the in-hospital survivor group (P < 0.001), whereas systemic infections without a clear primary site were less common (p = 0.001). Regarding pathogen type, no significant difference in overall bacterial infections between groups was observed; however, the non-survivor group had a higher proportion of gram-positive bacterial infections (p = 0.002), while in-hospital survivors had more gram-negative bacterial infections (p = 0.001). Fungal infections were more common in the non-survivor group than in the in-hospital survivor group (p = 0.035). The prevalence of atypical bacteria, viruses, tuberculosis, or multidrug-resistant pathogens did not differ significantly between the in-hospital survivor and non-survivor groups.

**Table 2:** Infection site and pathogens

Characteristics	All patients	Survivor	Non-survivor	P-value
Patients (n)	3733	2606	1127	
Suspected infection site of sepsis				
Pulmonary	1950 (52.2)	1260 (48.3)	690 (61.2)	< 0.001
Abdominal	783 (21.0)	568 (21.8)	215 (19.1)	0.061
Urinary	1,013 (27.1)	800 (30.7)	213 (18.9)	< 0.001
Skin/soft tissue	90 (2.4)	60 (2.3)	30 (2.7)	0.511
Catheter-related	17 (0.5)	12 (0.5)	5 (0.4)	0.944
Systemic infection without clear primary site of infection	232 (6.2)	139 (5.3)	93 (8.3)	0.001
Neurologic	19 (0.5)	14 (0.5)	5 (0.4)	0.712
Type of pathogen				
Bacteria	2,228 (59.7)	1,574 (60.4)	654 (58.0)	0.176
Gram positive bacteria	722 (19.3)	470 (18.0)	252 (22.4)	0.002
Gram negative bacteria	1,762 (47.2)	1,276 (49.0)	486 (43.1)	0.001
Atypical bacteria	13 (0.3)	8 (0.3)	5 (0.4)	0.515
Virus	47 (1.3)	28 (1.1)	19 (1.7)	0.124
Fungus	121 (3.2)	74 (2.8)	47 (4.2)	0.035
Tuberculosis	26 (0.7)	16 (0.6)	10 (0.9)	0.357
Others	8 (0.2)	7 (0.3)	1 (0.1)	0.275
MDR pathogens	996 (26.7)	690 (26.5)	306 (27.2)	0.669

Data are presented as mean ± standard deviation or number (%), unless otherwise indicated

### Interventions and outcomes of the patients

Supplementary Table 1 shows the interventions performed on the patients. ICU admission was more frequent in the non-survivor group than in the in-hospital survivor group (46.1% vs. 31.3%, p < 0.001). Invasive mechanical ventilation, high-flow nasal cannula (HFNC), and continuous renal replacement therapy (CRRT) were also more frequently used in the non-survival group. Additionally, life-sustaining treatment issues were significantly more common in the non-survival group (p < 0.001).

Supplementary Table 2 presents the clinical outcomes of the two groups, the in-hospital survivor group had longer hospital stays (p < 0.001), and no significant difference was observed in ICU stays (p = 0.441).

### Compliance for each *sepsis* bundle

Table 3 shows the compliance for each sepsis bundle. Compliance with the 1-h bundle did not differ significantly between the in-hospital survivor and non-survivor groups. Notably, lactate measurement within 1 h was higher in the non-survivor group than in the in-hospital survivor group (p = 0.026). Fluid therapy compliance was nearly universal; however, it was significantly higher in the in-hospital survivor group at 1 (p = 0.001), 3 h (p = 0.033) and 6 h (p = 0.017) than in the non-survivor group. Vasopressor use at 1, 3 and 6 h was significantly higher in the in-hospital survivor group than in the non-survivor group (p < 0.001). In the 3-h bundle, the non-survivor group had higher compliance with broad-spectrum antibiotics (p = 0.002) and lactate measurement within 3 h (p = 0.029) than the in-hospital survivor group. Compliance with the 6-h bundle showed no significant difference between groups, although lactate remeasurement was more adhered to in the non-survivor group than in the in-hospital survivor group (p = 0.025). Appropriateness of initial empiric antibiotics within 24 h was higher in the in-hospital survivor group than in the non-survivor group (p < 0.001).

**Table 3:** Compliance of each sepsis bundle

Characteristics	All patients	Survivor	Non-survivor	P-value
Patients (n)	3733	2606	1127	
1-h bundle	593 (15.9)	404 (15.5)	189 (16.8)	0.331
1-h Measurement of lactate	3,120 (83.6)	2,155 (82.7)	965 (85.6)	0.026
1-h Blood culture	2,437 (65.3)	1,713 (65.7)	724 (64.2)	0.380
1-h broad spectrum antibiotics	747 (20.0)	508 (19.5)	239 (21.2)	0.230
1-h fluid therapy	3,667 (98.2)	2,572 (98.7)	1,095 (97.2)	0.001
1-h use of vasopressor	3,464 (92.8)	2,472 (94.9)	992 (88.0)	< 0.001
3-h bundle	2,250 (60.3)	1,529 (58.7)	721 (64.0)	0.002
3-h Measurement of lactate	3,480 (93.2)	2,414 (92.6)	1,066 (94.6)	0.029
3-h Blood culture	3,304 (88.5)	2,304 (88.4)	1,000 (88.7)	0.779
3-h broad spectrum antibiotics	2,493 (66.8)	1,700 (65.2)	793 (70.4)	0.002
3-h fluid therapy	3,691 (98.9)	2,583 (99.1)	1,108 (98.3)	0.033
3-h use of vasopressor	3,602 (96.5)	2,536 (97.3)	1,066 (94.6)	< 0.001
6-h bundle	3,118 (83.5)	2,164 (83.0)	954 (84.6)	0.223
6-h Measurement of lactate	3,530 (94.6)	2,450 (94.0)	1,080 (95.8)	0.025
6-h Blood culture	3,581 (95.9)	2,506 (96.2)	1,075 (95.4)	0.270
6-h broad spectrum antibiotics	3,388 (90.8)	2,357 (90.4)	1,031 (91.5)	0.315
6-h fluid therapy	3,698 (99.1)	2,588 (99.3)	1,110 (98.5)	0.017
6-h use of vasopressor	3,657 (98.0)	2,567 (98.5)	1,090 (96.7)	< 0.001
Appropriateness of initial empiric antibiotics (within 24 h)	3,201 (85.7)	2,275 (87.3)	926 (82.2)	< 0.001

Data are presented as median and interquartile range, unless otherwise indicated

### Factors associated with in-hospital mortality

Table 4 presents the factors associated with in-hospital mortality including patient baseline characteristics, infection site and pathogens, interventions and sepsis bundle compliance assessed through Cox regression analysis. In the multivariable analysis, the following factors were significantly associated with higher in-hospital mortality: older age (HR, 1.019; 95% CI 1.002–1.036; p = 0.026), higher Clinical Frailty Scale (HR, 1.060; 95% CI 1.014–1.108; p = 0.010) and SOFA score (HR, 1.083; 95% CI, 1.059–1.108; p < 0.001), chronic obstructive pulmonary disease (HR, 1.281; 95% CI 1.018–1.611; p = 0.035). Conversely, cerebrovascular disease (HR, 0.815; 95% CI 0.686–0.968; p = 0.020), urinary tract infection (HR, 0.728; 95% CI 0.603–0.879; p < 0.001), and admission to the ICU (HR, 0.818; 95% CI 0.674–0.993; p = 0.043) were associated with lower in-hospital mortality.

**Table 4:** Univariate and multivariable Cox regression analysis addressing the factors for In-hospital mortality

	Univariate analysis			Multivariable analysis		
	HR	95% CI	*P*-value	HR	95% CI	*P*-value
Age, yr	1.034	1.019–1.049	< 0.001	1.019	1.002–1.036	0.026
Male	0.940	0.828–1.067	0.338			
Body mass index, kg/m^2^	0.973	0.953–0.989	0.001	0.994	0.977–1.011	0.488
Charlson comorbidity index	1.051	1.023–1.080	< 0.001	1.008	0.967–1.051	0.699
Clinical frailty scale	1.116	1.071–1.162	< 0.001	1.060	1.014–1.108	0.010
SOFA score	1.148	1.126–1.170	< 0.001	1.083	1.059–1.108	< 0.001
Underlying disease						
Solid tumor	1.220	1.056–1.410	0.007	1.141	0.932–1.397	0.202
Hematologic malignancy	1.972	1.447–2.687	< 0.001	1.366	0.979–1.904	0.066
Heart failure	1.188	0.970–1.454	0.095	0.968	0.777–1.206	0.774
Chronic obstructive pulmonary disease	1.319	1.073–1.621	0.009	1.281	1.018–1.611	0.035
Cerebrovascular disease	0.829	0.714–0.963	0.014	0.815	0.686–0.968	0.020
Suspected infection site of sepsis						
Pulmonary	1.515	1.331–1.724	< 0.001	1.087	0.916–1.290	0.339
Abdominal	0.907	0.774–1.063	0.229			
Urinary	0.633	0.540–0.741	< 0.001	0.728	0.603–0.879	< 0.001
Systemic infection without clear primary site of infection	1.398	1.096–1.783	0.007	1.038	0.775–1.389	0.804
Type of pathogen						
Gram positive bacteria	0.898	0.774–1.042	0.157			
Gram negative bacteria	1.214	1.069–1.379	0.003	0.926	0.799–1.073	0.308
Fungus	0.867	0.645–1.166	0.346			
Admission to the ICU	1.139	1.002–1.294	0.046	0.818	0.674–0.993	0.043
Interventions in the ICU						
Invasive mechanical ventilation	1.754	1.535–2.004	< 0.001	1.404	1.155–1.706	< 0.001
NIV	0.808	0.519–1.260	0.347			
HFNC	1.032	0.875–1.218	0.705			
Continuous renal replacement therapy	2.206	1.895–2.570	< 0.001	1.622	1.343–1.959	< 0.001
Life sustaining treatment issue	8.277	6.996–9.792	< 0.001	6.665	5.564–7.984	< 0.001
Sepsis bundle						
1-h Measurement of lactate	1.252	1.051–1.492	0.012	0.995	0.785–1.262	0.969
1-h fluid therapy	0.562	0.383–0.824	0.003	0.503	0.265–0.955	0.036
1-h use of vasopressor	0.575	0.465–0.711	< 0.001	0.622	0.452–0.856	0.004
3-h bundle	1.201	1.054–1.367	0.006	1.116	0.802–1.554	0.513
3-h Measurement of lactate	1.483	1.142–1.926	0.003	1.139	0.614–2.110	0.680
3-h broad spectrum antibiotics	0.889	0.775–1.018	0.089	0.875	0.634–1.207	0.415
3-h fluid therapy	0.704	0.429–1.155	0.165			
3-h use of vasopressor	0.649	0.475–0.887	0.007	1.198	0.667–2.150	0.545
6-h Measurement of lactate	1.492	1.112–2.000	0.008	0.807	0.429–1.516	0.504
6-h fluid therapy	0.625	0.369–1.060	0.081	1.203	0.500–2.893	0.680
6-h use of vasopressor	0.585	0.390–0.878	0.010	1.052	0.535–2.067	0.884
Appropriateness of initial empiric therapy (within 24 h)	0.706	0.599–0.831	< 0.001	0.836	0.698–1.002	0.053

HR; Hazard ratio, CI; Confidence interval, SOFA; Sequential Organ Failure Assessment, ICU; Intensive care unit, HFNC; high flow nasal cannula

**Fig. 2:**
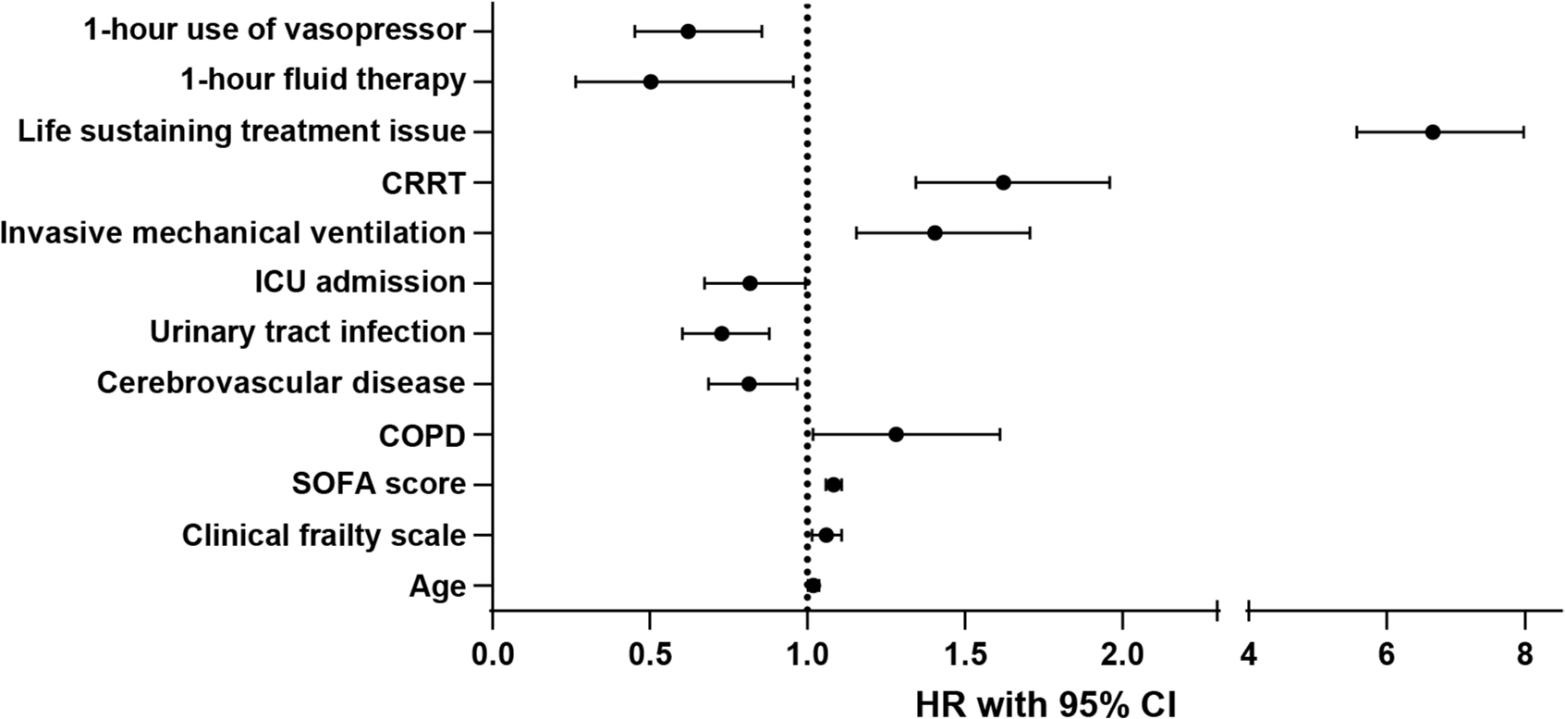
Factors associated with in-hospital mortality using Cox regression analysis. The data obtained from SPSS was reconstructed into graphs using GraphPad Prism. HR, Hazard ratio; CI, Confidence interval; COPD, Chronic obstructive pulmonary disease; CRRT, Continuous renal replacement therapy

Additionally, invasive mechanical ventilation (HR, 1.404; 95% CI 1.155–1.706; p < 0.001), CRRT use (HR, 1.622; 95% CI 1.343–1. 959; p < 0.001), issue of life-sustaining treatment (HR, 6.665; 95% CI 5.564–7.984; p < 0.001) were strongly associated with increased mortality. Timely interventions within the sepsis bundle, such as fluid therapy within 1 h (HR, 0.503; 95% CI, 0.265–0.955; p = 0.036) and vasopressor use within 1 h (HR, 0.655; 95% CI 0.522–0.822; p < 0.001), were associated with reduced in-hospital mortality. Figure 2**,** a Forest plot, illustrates the association between these significant factors and in-hospital mortality.

Furthermore, we used the Clinical Frailty Scale (CFS) because it is a reliable predictor of outcomes in old age patients by reflecting functional status, particularly in critical illnesses. We adjusted for disease severity using the SOFA score to assess organ dysfunction. Notably, patients with a frailty score of 8 or higher had a significantly increased mortality rate compared to those with a frailty score of 3 or lower (p = 0.01, HR = 1.675), confirming the importance of frailty in predicting outcomes in older patients (Supplementary Fig. 1).

### Subgroup analysis according to LST

Given the strong association between life-sustaining treatment (LST) and in-hospital mortality, as reflected by the high hazard ratio (HR, 6.665; 95% CI 5.564–7.984; p < 0.001), we conducted a subgroup analysis based on LST status. Supplementary Table 3 presents the baseline characteristics, infection sources, and sepsis bundle compliance differences between survivor and non-survivor group according to LST status. Subsequently, significant factors were analyzed through Cox regression analysis (Supplementary Table 4, 5, and Supplementary Fig. 2). Regardless of LST status, CRRT, MV, and SOFA score were statistically significant factors associated with increased in-hospital mortality. Additionally, in the LST group, early vasopressor administration and ICU admission were found to have the potential to improve survival.

## Discussion

This study’s results provide insight into factors that influence survival outcomes and sepsis bundle compliance in very old patients with sepsis. Several patient characteristics and compliance with sepsis bundle were associated with in-hospital mortality.

In our study, the non-survivor group was more likely to be male, have a lower BMI, be more frail, and have higher SOFA scores. In addition, comorbidities, except for cerebrovascular disease, were more common in the non-survivor group. These findings are consistent with previous studies suggesting that these characteristics may negatively impact survival. For instance, previous research has reported higher mortality in males with sepsis [16], and frailty has been shown to increase the mortality [17, 18], especially in older population. Several comorbidities have also been more prevalent in non-survivors [19, 20], suggesting a complex interaction between pre-existing health conditions and survival outcomes in the older sepsis patients.

Interestingly, cognitive disorders, cerebrovascular disease, and living in a nursing home are typically considered poor prognostic factors in sepsis patients. However, in our study, neither cognitive disorders nor living in a nursing home showed statistically significant associations with outcomes, and cerebrovascular disease was more common among survivors. Moreover, the Charlson Comorbidity Index, which reflects comorbidity burden, was not significant in the multivariable Cox regression analysis (p = 0.699). In contrast, the Clinical Frailty Scale (HR 1.060, p = 0.01), reflecting performance status, and the SOFA score (HR 1.083, p < 0.001), indicating disease severity, were statistically significant. This suggests that in very old sepsis patients, performance status, which reflects the patient's functional capacity and resilience to recover from severe illnesses, plays a more critical role in prognosis than the burden of comorbidities. Additionally, most studies on older adults have been conducted in populations aged 65 and above, they may not fully capture the distinct characteristics of very old patients.

In our cohort of very old sepsis patients, pulmonary infections were the most common, followed by urinary tract and abdominal infections. The non-survivor group had a higher prevalence of pulmonary and systemic infections without a clear primary site. This aligns with previous studies identifying respiratory infections as a major cause of sepsis [9, 21], often leading to poorer outcomes [22, 23] due to the high risk of rapid deterioration and respiratory failure [24]. Regarding pathogens, our findings of a higher incidence of gram-positive bacteria and fungi in the non-survivor group are consistent with previous research [25, 26]. The increased prevalence of these pathogens may be linked to their role in more severe forms of sepsis and their association with a higher risk of mortality, particularly in immunocompromised or frail older individuals [9, 27]. These findings further highlight the need for vigilant monitoring of specific pathogens and infection sites in this population.

There is mixed evidence regarding the effectiveness of bundle therapy for sepsis. Some studies have shown that the 1-h sepsis bundle positively affect patient prognosis [13]. Umemura et al. [14] reported a significant difference in in-hospital mortality between patients who received the 1-h sepsis bundle (18.0%) and those who did not (30.3%) (p = 0.054). However, other studies, such as Rhee et al. [28], found that while sepsis bundle increased lactate measurement within 1 h, it did not improve patient outcomes. Similar mixed results were observed with the 3- and 6-h sepsis bundles improves patient outcomes [29–31], while others show no significant effect [32, 33].

These findings suggest that achieving compliance with the sepsis bundles can be challenging, potentially due to difficulties in early sepsis recognition [34, 35]. Consequently, various strategies have been implemented to increase bundle compliance and enhance patient outcomes [36]. These include efforts to enhance early recognition of sepsis [37], process changes to streamline the use of sepsis bundle for timely interventions [38], and educational programs to raise awareness and emphasize the importance of adhering to sepsis management protocols [39]. Such efforts aim to overcome barriers to bundle adherence and improve outcomes for sepsis patients. For example, a systematic review and meta-analysis by Daimani et al. [40] found that performance improvement programs were directly associated with increased adherence to resuscitation and sepsis management bundles, significantly reducing mortality in sepsis patients.

Through multivariable analysis, we identified several factors associated with in-hospital mortality. Compliance with the 1-h fluid therapy and 1-h vasopressor use was significantly associated with reduced in-hospital mortality. Older age, higher clinical frailty scale, and SOFA score were predictive of worse outcomes, reinforcing the need for age-appropriate treatment strategies. Additionally, underlying medical conditions such as chronic obstructive pulmonary disease were predictors of mortality, suggesting the importance of integrated management for sepsis and comorbidities. These findings are consistent with previous studies that have highlighted similar factors influencing sepsis outcomes. Previous studies have demonstrated the impact of age-related increases in mortality risk [8, 41], pre-existing chronic conditions [42], frailty [17, 43], and high SOFA scores [44] on patient outcomes. Invasive interventions in the ICU [45], such as mechanical ventilation and CRRT, as well as life-sustaining treatment issues [43, 46], are also strongly associated with in-hospital mortality.

However, limited data exists on very old patients, particularly with regarding adherence to sepsis bundle therapy. Previous studies have shown mixed results on the overall effectiveness of the 1-h bundle adherence [13–15, 47], with varying outcomes for the 3- and 6-h bundles [8, 48, 49]. Our findings suggests that, in very old populations, each component of the bundle may not equally contribute to outcomes. Therefore, it is important to identify which interventions, when implemented rapidly, can significantly influence sepsis outcomes in these patients.

Vasopressor use in managing sepsis addresses one of the major complications, septic shock [50], and has long been regarded as a marker of illness severity. Vasopressors, such as norepinephrine, dopamine, and vasopressin, work by increasing vascular tone, thereby raising blood pressure and improving perfusion to vital organs [51]. Certain studies suggest the delays in administrating vasopressors, obtaining blood cultures, and administering antibiotics can exacerbate outcomes in sepsis patients [14, 15]. However, other research indicates that the timing of vasopressor initiation may not necessarily correlate with prognosis [47]. Additionally, the effect of vasopressors on the prognosis in elderly patients remains uncertain. Our study found that vasopressor use within the 1-h sepsis bundle was associated with reduced mortality, even when analyzed alongside the patient's underlying conditions. Furthermore, fluid resuscitation within the first hour also demonstrated a significant impact on reducing mortality, with the p-value of 0.036 in the multivariable Cox regression analysis. These findings underscore the importance of optimizing sepsis bundle performance, particularly in relation to organ perfusion, for elderly patients. Given the increasing number of very old patients, further studies examining the efficacy and feasibility of sepsis bundles tailored to the specific challenges of this population are necessary. Addressing this gap is essential for developing targeted interventions to improve outcomes for this vulnerable population.

Considering the high hazard ratio for life-sustaining treatment (LST) and its strong association with in-hospital mortality (HR, 6.665; 95% CI, 5.564–7.984; p < 0.001), we further explored its impact by conducting a subgroup analysis based on LST status. This analysis provided additional insights into the management of sepsis in very old patients with and without LST limitations. Our analysis demonstrated that, regardless of LST status, several factors, such as CRRT, mechanical ventilation (MV), and higher SOFA scores, were consistently associated with increased in-hospital mortality. These results align with the existing literature, which highlights the burden of organ dysfunction and the role of invasive interventions in influencing outcomes in sepsis patients. Interestingly, early vasopressor administration and ICU admission were identified as potentially beneficial interventions only in the group with life-sustaining treatment (LST) limitations, while no such benefit was observed in the group without LST limitations. This finding suggests that in patients without LST limitations who have full access to intensive interventions, standard bundle elements may not be as critical in influencing outcomes. Instead, other clinical factors, such as infection type and comorbidity status, may have a greater impact on prognosis. Conversely, this result indicates that even among patients with LST limitations, proactive management strategies may still improve survival. It challenges the assumption that patients with LST limitations may not benefit from aggressive interventions and underscores the importance of careful clinical decision-making in very old sepsis patients. Ensuring that appropriate treatment options are considered, regardless of LST status.

This study has several limitations. First, as an observational study, residual confounding cannot be completely ruled out despite adjustment for various variables. However, we tried to ensure a robust sample size and used rigorous statistical methods to enhance credibility of our findings. Second, although real-time screening and active data acquisition were performed prospectively during the study period, this study relies on data extraction from medical records, resembling a registry-based study. This may present challenges related to the accuracy and completeness of healthcare professionals' documentation. Recognizing these limitations, we employed strict record-keeping protocols and experienced researchers to minimize potential bias. Third, differences in patient demographics and hospital practices highlight the inherent limitations of the study. Hospital heterogeneity means that baseline characteristics and sepsis outcomes may vary due to site-specific factors, potentially affecting the generalizability of the results. Variations in epidemiologic patterns and identified risk factors may also depend on the demographics and treatment protocols of the participating institutions.

## Conclusions

In conclusion, our findings suggest that early intervention and compliance with the sepsis bundle components, particularly those related to organ perfusion, significantly improve outcomes in very old patients. Moreover, the patient's underlying condition and functional status are key determinants of prognosis well. These results highlight the importance of clinical vigilance and comprehensive care tailored to the unique characteristics of very old patients to optimize survival in this vulnerable population.

## Supplementary Information

Supplementary material 1:  Supplementary Figure 1. Cox regression survival curve adjusted for SOFA severity score for the whole cohort, grouped by Clinical Frailty Scale (CFS). The data obtained from SPSS was reconstructed into graphs using illustrator. HR, Hazard ratio; CI, Confidence interval.

Supplementary material 2: Supplementary Figure 2. Factors associated with in-hospital mortality according to LST using Cox regression analysis. The data obtained from SPSS was reconstructed into graphs using GraphPad Prism. HR, Hazard ratio; CI, Confidence interval; COPD, Chronic obstructive pulmonary disease; CRRT, Continuous renal replacement therapy.

Supplementary material 3.

## Data Availability

The data supporting the findings of this study are available upon request from the corresponding author. The data are not publicly available because of privacy or ethical restrictions.

## Abbreviations

ICU: Intensive care unit
SSC: Surviving sepsis campaign
SOFA: Sequential organ failure assessment
ECOG: Eastern cooperative oncology group
HR: Hazard ratio
CI: Confidence interval
HFNC: High-flow nasal cannula
CRRT: Continuous renal replacement therapy

## References

[1] Chen LK. Urbanization and population aging: converging trends of demographic transitions in modern world. Arch Gerontol Geriatr. 2022;101: 104709. 35489310 10.1016/j.archger.2022.104709

[2] Kim KW, Kim OS. Super aging in South Korea unstoppable but mitigatable: a sub-national scale population projection for best policy planning. Spatial Demography. 2020;8(2):155–73. 34222615 10.1007/s40980-020-00061-8 PMC8248505

[3] Cobert J, Jeon SY, Boscardin J, Chapman AC, Ferrante LE, Lee S, Smith AK. Trends in geriatric conditions among older adults admitted to US ICUs between 1998 and 2015. Chest. 2022;161(6):1555–65. 35026299 10.1016/j.chest.2021.12.658 PMC9248079

[4] Garland A, Olafson K, Ramsey CD, Yogendran M, Fransoo R. Epidemiology of critically ill patients in intensive care units: a population-based observational study. Crit Care. 2013;17(5):R212. 24079640 10.1186/cc13026 PMC4056438

[5] Haas LE, Karakus A, Holman R, Cihangir S, Reidinga AC, de Keizer NF. Trends in hospital and intensive care admissions in the Netherlands attributable to the very elderly in an ageing population. Crit Care. 2015;19:353. 26423744 10.1186/s13054-015-1061-z PMC4588268

[6] Singer M, Deutschman CS, Seymour CW, Shankar-Hari M, Annane D, Bauer M, et al. The third international consensus definitions for sepsis and septic shock (sepsis-3). JAMA. 2016;315(8):801–10. 26903338 10.1001/jama.2016.0287 PMC4968574

[7] Li A, Ling L, Qin H, Arabi YM, Myatra SN, Egi M, et al. Epidemiology, management, and outcomes of sepsis in ICUs among countries of differing national wealth across Asia. Am J Respir Crit Care Med. 2022;206(9):1107–16. 35763381 10.1164/rccm.202112-2743OC

[8] Martin-Loeches I, Guia MC, Vallecoccia MS, Suarez D, Ibarz M, Irazabal M, et al. Risk factors for mortality in elderly and very elderly critically ill patients with sepsis: a prospective, observational, multicenter cohort study. Ann Intensive Care. 2019;9(1):26. 30715638 10.1186/s13613-019-0495-x PMC6362175

[9] Rowe TA, McKoy JM. Sepsis in older adults. Infect Dis Clin North Am. 2017;31(4):731–42. 29079157 10.1016/j.idc.2017.07.010

[10] Turnbull AE, Lau BM, Ruhl AP, Mendez-Tellez PA, Shanholtz CB, Needham DM. Age and decisions to limit life support for patients with acute lung injury: a prospective cohort study. Crit Care. 2014;18(3):R107. 24886945 10.1186/cc13890 PMC4075260

[11] Evans L, Rhodes A, Alhazzani W, Antonelli M, Coopersmith CM, French C, et al. Surviving sepsis campaign: international guidelines for management of sepsis and septic shock 2021. Crit Care Med. 2021;49(11):e1063–143. 34605781 10.1097/CCM.0000000000005337

[12] Daniels R, Foot E, Pittaway S, Urzi S, Favry A, Miller M. Survey of adherence to sepsis care bundles in six European countries shows low adherence and possible patient risk. BMJ Open Qual. 2023;12(2):e002304. 37286298 10.1136/bmjoq-2023-002304 PMC10254959

[13] Townsend SR, Phillips GS, Duseja R, Tefera L, Cruikshank D, Dickerson R, et al. Effects of compliance with the early management bundle (SEP-1) on mortality changes among medicare beneficiaries with sepsis: a propensity score matched cohort study. Chest. 2022;161(2):392–406. 34364867 10.1016/j.chest.2021.07.2167

[14] Umemura Y, Abe T, Ogura H, Fujishima S, Kushimoto S, Shiraishi A, et al. Hour-1 bundle adherence was associated with reduction of in-hospital mortality among patients with sepsis in Japan. PLoS ONE. 2022;17(2): e0263936. 35157744 10.1371/journal.pone.0263936 PMC8843226

[15] Ko BS, Choi SH, Shin TG, Kim K, Jo YH, Ryoo SM, et al. Impact of 1-Hour bundle achievement in septic shock. J Clin Med. 2021;10(3):527. 33540513 10.3390/jcm10030527 PMC7867161

[16] Ko RE, Kang D, Cho J, Na SJ, Chung CR, Lim SY, et al. Influence of gender on age-associated in-hospital mortality in patients with sepsis and septic shock: a prospective nationwide multicenter cohort study. Crit Care. 2023;27(1):229. 37303037 10.1186/s13054-023-04515-5 PMC10257805

[17] Dong J, Chen R, Song X, Guo Z, Sun W. Quality of life and mortality in older adults with sepsis after one-year follow up: a prospective cohort study demonstrating the significant impact of frailty. Heart Lung. 2023;60:74–80. 36931009 10.1016/j.hrtlng.2023.03.002

[18] Bruno RR, Wernly B, Bagshaw SM, van den Boogaard M, Darvall JN, De Geer L, et al. The clinical frailty scale for mortality prediction of old acutely admitted intensive care patients: a meta-analysis of individual patient-level data. Ann Intensive Care. 2023;13(1):37. 37133796 10.1186/s13613-023-01132-x PMC10155148

[19] Thomas-Rüddel DO, Fröhlich H, Schwarzkopf D, Bloos F, Riessen R. Sepsis and underlying comorbidities in intensive care unit patients: analysis of the cause of death by different clinicians-a pilot study. Med Klin Intensivmed Notfmed. 2024;119(2):123–8. 37380812 10.1007/s00063-023-01037-4 PMC10901974

[20] Yang Y, Yang KS, Hsann YM, Lim V, Ong BC. The effect of comorbidity and age on hospital mortality and length of stay in patients with sepsis. J Crit Care. 2010;25(3):398–405. 19836195 10.1016/j.jcrc.2009.09.001

[21] Sousa ÁFL, Queiroz A, Oliveira LB, Moura LKB, Andrade D, Watanabe E, Moura MEB. Deaths among the elderly with ICU infections. Rev Bras Enferm. 2017;70(4):733–9. 28793102 10.1590/0034-7167-2016-0611

[22] Motzkus CA, Luckmann R. Does infection site matter? A systematic review of infection site mortality in sepsis. J Intensive Care Med. 2017;32(8):473–9. 26880006 10.1177/0885066615627778

[23] Collaborators GAR. Global mortality associated with 33 bacterial pathogens in 2019: a systematic analysis for the global burden of disease study 2019. Lancet. 2022;400(10369):2221–48. 36423648 10.1016/S0140-6736(22)02185-7 PMC9763654

[24] Luyt CE, Bouadma L, Morris AC, Dhanani JA, Kollef M, Lipman J, et al. Pulmonary infections complicating ARDS. Intensive Care Med. 2020;46(12):2168–83. 33175277 10.1007/s00134-020-06292-z PMC7656898

[25] Singh N, Puri S, Anshul Kumar S, Pahuja H, Kalia R, Arora R. Risk factors and outcome analysis of gram-positive bacteremia in critically ill patients. Cureus. 2023;15(3):e36585. 37097814 10.7759/cureus.36585 PMC10122440

[26] Yang SP, Chen YY, Hsu HS, Wang FD, Chen LY, Fung CP. A risk factor analysis of healthcare-associated fungal infections in an intensive care unit: a retrospective cohort study. BMC Infect Dis. 2013;13:10. 23298156 10.1186/1471-2334-13-10 PMC3548709

[27] Ibarz M, Haas LEM, Ceccato A, Artigas A. The critically ill older patient with sepsis: a narrative review. Ann Intensive Care. 2024;14(1):6. 38200360 10.1186/s13613-023-01233-7 PMC10781658

[28] Rhee C, Yu T, Wang R, Kadri SS, Fram D, Chen HC, Klompas M. Association between implementation of the severe sepsis and septic shock early management bundle performance measure and outcomes in patients with suspected sepsis in US hospitals. JAMA Netw Open. 2021;4(12): e2138596. 34928358 10.1001/jamanetworkopen.2021.38596 PMC8689388

[29] Memon JI, Rehmani RS, Alaithan AM, El Gammal A, Lone TM, Ghorab K, Abdulbasir A. Impact of 6-hour sepsis resuscitation bundle compliance on hospital mortality in a saudi hospital. Crit Care Res Pract. 2012;2012: 273268. 23082248 10.1155/2012/273268 PMC3469084

[30] Gao F, Melody T, Daniels DF, Giles S, Fox S. The impact of compliance with 6-hour and 24-hour sepsis bundles on hospital mortality in patients with severe sepsis: a prospective observational study. Crit Care. 2005;9(6):R764-770. 16356225 10.1186/cc3909 PMC1414020

[31] Levy MM, Gesten FC, Phillips GS, Terry KM, Seymour CW, Prescott HC, et al. Mortality changes associated with mandated public reporting for sepsis. The results of the New York state initiative. Am J Respir Crit Care Med. 2018;198(11):1406–12. 30189749 10.1164/rccm.201712-2545OC PMC6290949

[32] August BA, Griebe KM, Stine JJ, Hauser CD, Hunsaker T, Jones MC, et al. Evaluating the impact of severe sepsis 3-hour bundle compliance on 28-day in-hospital mortality: a propensity adjusted, nested case-control study. Pharmacotherapy. 2022;42(8):651–8. 35774011 10.1002/phar.2715

[33] Deis AS, Whiles BB, Brown AR, Satterwhite CL, Simpson SQ. Three-hour bundle compliance and outcomes in patients with undiagnosed severe sepsis. Chest. 2018;153(1):39–45. 28987477 10.1016/j.chest.2017.09.031 PMC6689078

[34] Roberts N, Hooper G, Lorencatto F, Storr W, Spivey M. Barriers and facilitators towards implementing the Sepsis Six care bundle (BLISS-1): a mixed methods investigation using the theoretical domains framework. Scand J Trauma Resusc Emerg Med. 2017;25(1):96. 28927439 10.1186/s13049-017-0437-2 PMC5606082

[35] Adams R, Henry KE, Sridharan A, Soleimani H, Zhan A, Rawat N, et al. Prospective, multi-site study of patient outcomes after implementation of the TREWS machine learning-based early warning system for sepsis. Nat Med. 2022;28(7):1455–60. 35864252 10.1038/s41591-022-01894-0

[36] Schinkel M, Nanayakkara PWB, Wiersinga WJ. Sepsis performance improvement programs: from evidence toward clinical implementation. Crit Care. 2022;26(1):77. 35337358 10.1186/s13054-022-03917-1 PMC8951662

[37] Warttig S, Alderson P, Evans DJ, Lewis SR, Kourbeti IS, Smith AF. Automated monitoring compared to standard care for the early detection of sepsis in critically ill patients. Cochrane Database Syst Rev. 2018;6(6):Cd012404. 29938790 10.1002/14651858.CD012404.pub2 PMC6353245

[38] Girardis M, Rinaldi L, Donno L, Marietta M, Codeluppi M, Marchegiano P, Venturelli C. Effects on management and outcome of severe sepsis and septic shock patients admitted to the intensive care unit after implementation of a sepsis program: a pilot study. Crit Care. 2009;13(5):R143. 19728879 10.1186/cc8029 PMC2784353

[39] van Zanten AR, Brinkman S, Arbous MS, Abu-Hanna A, Levy MM, de Keizer NF. Guideline bundles adherence and mortality in severe sepsis and septic shock. Crit Care Med. 2014;42(8):1890–8. 24670937 10.1097/CCM.0000000000000297

[40] Damiani E, Donati A, Serafini G, Rinaldi L, Adrario E, Pelaia P, et al. Effect of performance improvement programs on compliance with sepsis bundles and mortality: a systematic review and meta-analysis of observational studies. PLoS ONE. 2015;10(5): e0125827. 25946168 10.1371/journal.pone.0125827 PMC4422717

[41] Nasa P, Juneja D, Singh O, Dang R, Arora V. Severe sepsis and its impact on outcome in elderly and very elderly patients admitted in intensive care unit. J Intensive Care Med. 2012;27(3):179–83. 21436163 10.1177/0885066610397116

[42] Haas LEM, Boumendil A, Flaatten H, Guidet B, Ibarz M, Jung C, et al. Frailty is associated with long-term outcome in patients with sepsis who are over 80 years old: results from an observational study in 241 European ICUs. Age Ageing. 2021;50(5):1719–27. 33744918 10.1093/ageing/afab036

[43] Guidet B, de Lange DW, Boumendil A, Leaver S, Watson X, Boulanger C, et al. The contribution of frailty, cognition, activity of daily life and comorbidities on outcome in acutely admitted patients over 80 years in European ICUs: the VIP2 study. Intensive Care Med. 2020;46(1):57–69. 31784798 10.1007/s00134-019-05853-1 PMC7223711

[44] Polok K, Fronczek J, Putowski Z, Czok M, Guidet B, Jung C, et al. Validity of the total SOFA score in patients ≥ 80 years old acutely admitted to intensive care units: a post-hoc analysis of the VIP2 prospective, international cohort study. Ann Intensive Care. 2023;13(1):98. 37798561 10.1186/s13613-023-01191-0 PMC10555975

[45] Vincent JL, Rello J, Marshall J, Silva E, Anzueto A, Martin CD, et al. International study of the prevalence and outcomes of infection in intensive care units. JAMA. 2009;302(21):2323–9. 19952319 10.1001/jama.2009.1754

[46] Boonmee P, Ruangsomboon O, Limsuwat C, Chakorn T. Predictors of mortality in elderly and very elderly emergency patients with sepsis: a retrospective study. West J Emerg Med. 2020;21(6):210–8. 33207168 10.5811/westjem.2020.7.47405 PMC7673873

[47] Ahn C, Yu G, Shin TG, Cho Y, Park S, Suh GY. Comparison of early and late norepinephrine administration in patients with septic shock: A systematic review and meta-analysis. Chest. 2024. Online ahead of print. 10.1016/j.chest.2024.05.042 38972348

[48] Venkatesh B, Schlapbach L, Mason D, Wilks K, Seaton R, Lister P, et al. Impact of 1-hour and 3-hour sepsis time bundles on patient outcomes and antimicrobial use: a before and after cohort study. Lancet Reg Health West Pac. 2022;18: 100305. 35024649 10.1016/j.lanwpc.2021.100305 PMC8654968

[49] de Groot B, Ansems A, Gerling DH, Rijpsma D, van Amstel P, Linzel D, et al. The association between time to antibiotics and relevant clinical outcomes in emergency department patients with various stages of sepsis: a prospective multi-center study. Crit Care. 2015;19(1):194. 25925412 10.1186/s13054-015-0936-3 PMC4440486

[50] Scheeren TWL, Bakker J, De Backer D, Annane D, Asfar P, Boerma EC, et al. Current use of vasopressors in septic shock. Ann Intensive Care. 2019;9(1):20. 30701448 10.1186/s13613-019-0498-7 PMC6353977

[51] Russell JA. Vasopressor therapy in critically ill patients with shock. Intensive Care Med. 2019;45(11):1503–17. 31646370 10.1007/s00134-019-05801-z

