# Supplementary figures and images for: Influence of underlying condition and performance of sepsis bundle in very old patients with sepsis: a nationwide cohort study
Source: Ann Intensive Care. 2024 Dec 4;14:179. doi: 10.1186/s13613-024-01415-x (PMC11618279; doi:10.1186/s13613-024-01415-x)

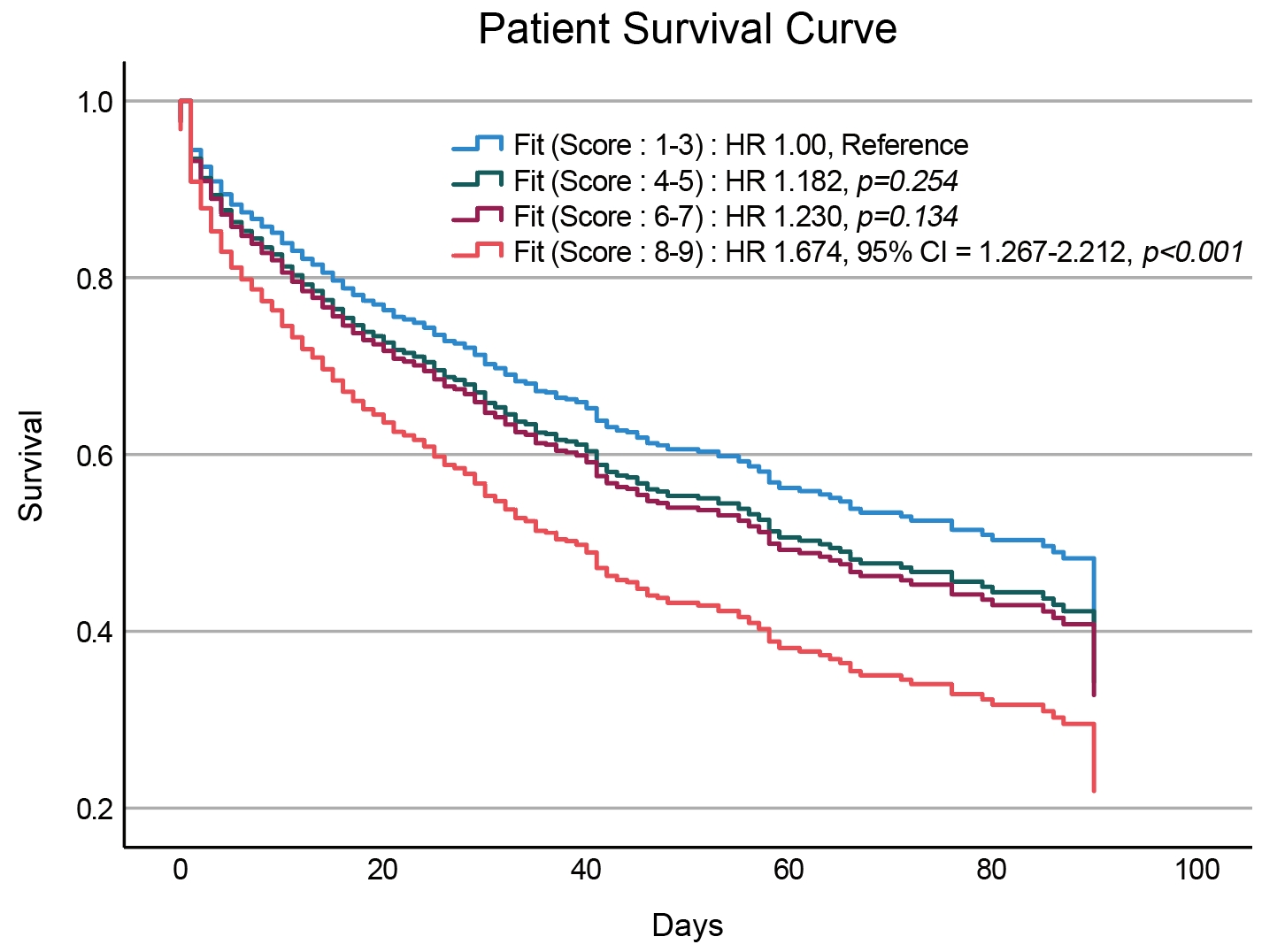

Supplement: Supplementary file 1 — Supplementary material 1: Supplementary Figure 1. Cox regression survival curve adjusted for SOFA severity score for the whole cohort, grouped by Clinical Frailty Scale (CFS). The data obtained from SPSS was reconstructed into graphs using illustrator. HR, Hazard ratio; CI, Confidence interval. [file 13613_2024_1415_MOESM1_ESM.jpg]

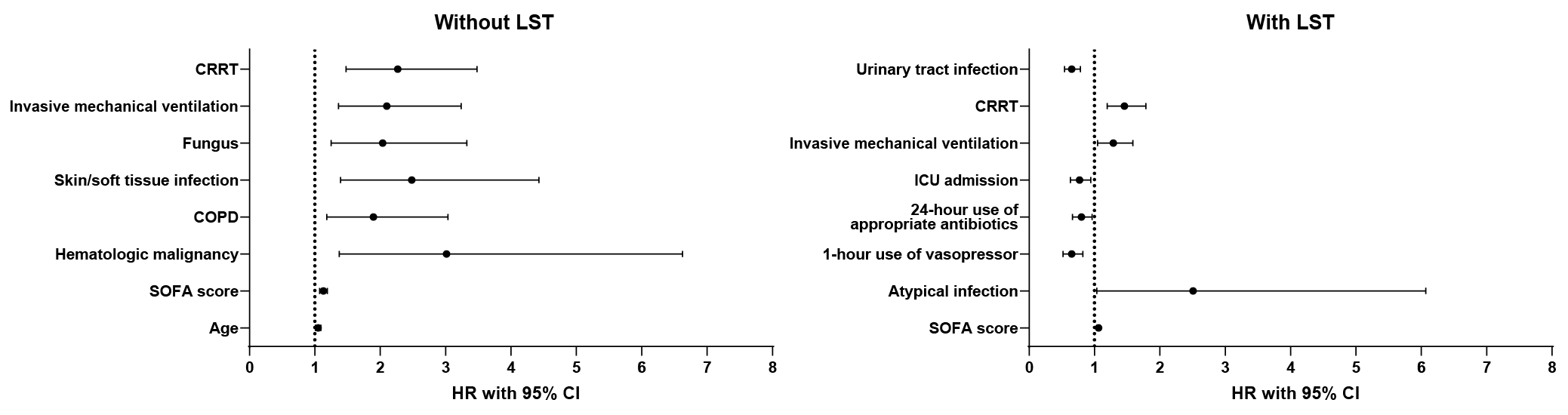

Supplement: Supplementary file 2 — Supplementary material 2: Supplementary Figure 2. Factors associated with in-hospital mortality according to LST using Cox regression analysis. The data obtained from SPSS was reconstructed into graphs using GraphPad Prism. HR, Hazard ratio; CI, Confidence interval; COPD, Chronic obstructive pulmonary disease; CRRT, Continuous renal replacement therapy. [file 13613_2024_1415_MOESM2_ESM.jpg]
