# Supplementary material for: Influence of underlying condition and performance of sepsis bundle in very old patients with sepsis: a nationwide cohort study
Source: Ann Intensive Care. 2024 Dec 4;14:179. doi: 10.1186/s13613-024-01415-x (PMC11618279; doi:10.1186/s13613-024-01415-x)
Supplement: Supplementary file 3 — Supplementary material 3. [file 13613_2024_1415_MOESM3_ESM.docx]

Supplementary Table 1. Interventions and ICU care in sepsis patients

| Characteristics | All patients | In-hospital Survivor | Non-survivor | P-value |
| --- | --- | --- | --- | --- |
| Admission to the ICU | 1,414 (37.9) | 894 (31.3) | 520 (46.1) | <0.001 |
| Interventions in the ICU |  |  |  |  |
| Invasive mechanical  ventilation | 645 (17.3) | 303 (11.6) | 342 (30.3) | <0.001 |
| NIV | 60 (1.6) | 40 (1.5) | 20 (1.8) | 0.593 |
| HFNC | 439 (11.8) | 266 (10.2) | 173 (15.4) | <0.001 |
| Continuous renal  replacement therapy | 331 (8.9) | 115 (4.4) | 216 (19.2) | <0.001 |
| ECMO | 3 (0.1) | 2 (0.1) | 1 (0.1) | 0.906 |
| Hemodialysis | 31 (0.8) | 20 (0.8) | 11 (1.0) | 0.519 |
| Life sustaining treatment issue | 1,603 (42.9) | 659 (25.3) | 944 (83.8) | <0.001 |

Data are presented as median and interquartile range or number (%), unless otherwise indicated.

ICU, intensive Care Unit; HFNC, high flow nasal cannula; ECMO, extracorporeal membrane oxygenation

Supplementary Table 2. Clinical outcomes of the patients

| Characteristics | All patients | In-hospital Survivor | Non-survivor | P-value |
| --- | --- | --- | --- | --- |
| Hospital stay, days | 12.0 (6.0 – 21.0) | 13.0 (8.0 – 23.0) | 5.0 (2.0 – 15.0) | <0.001 |
| ICU stay, days | 4.0 (2.0 – 9.0) | 4.0 (2.0 – 9.0) | 3.0 (1.0 – 9.0) | 0.441 |

Data are presented as median and interquartile range, unless otherwise indicated.

ICU, intensive Care Unit

Supplementary Table 3. Subgroup analysis based on life-sustaining treatment issue

| Characteristics | In-hospital Survivor without LST | Non-Survivor without LST | P-value | In-hospital Survivor  with LST | Non-Survivor with LST | P-value |
| --- | --- | --- | --- | --- | --- | --- |
| Patients (n) | 1947 | 183 |  | 659 | 944 |  |
| Age, yr | 84.72 ± 3.93 | 85.05 ± 3.98 | <0.028 | 86.26 ± 4.64 | 85.49 ± 4.383 | 0.047 |
| Male | 938 (48.2) | 105 (57.4) | 0.017 | 303 (46.0) | 500 (53) | 0.006 |
| Body mass index, kg/m^2^ | 21.56 ± 3.99 | 21.14 ± 3.56 | <0.085 | 20.50 ± 3.96 | 20.68 ± 3.90 | 0.561 |
| Resident of nursing care center | 300 (15.4) | 27 (14.8) | 0.814 | 169 (25.6) | 174 (18.4) | <0.001 |
| Charlson comorbidity  index | 6.3 ± 1.8 | 7.14 ± 2.5 | <0.001 | 6.5 ± 2.0 | 6.69 ± 2.2 | 0.004 |
| Clinical frailty scale | 5.9 ± 1.7 | 6.3 ± 1.6 | 0.128 | 6.7 ± 1.4 | 6.4 ± 1.6 | 0.001 |
| SOFA score | 5.5 ± 2.5 | 7.6 ± 3.6 | <0.001 | 6.5 ± 2.6 | 7.6 ± 3.2 | 0.001 |
| Underlying disease |  |  |  |  |  |  |
| Diabetes Mellitus | 698 (35.9) | 76 (41.5) | 0.127 | 207 (22.9) | 304 (32.2) | 0.738 |
| Solid tumor | 403 (20.7) | 44 (24) | 0.288 | 158 (24.0) | 271 (28.7) | 0.035 |
| Hematologic malignancy | 29 (1.5) | 8 (4.4) | 0.004 | 17 (2.6) | 38 (4) | 0.118 |
| Chronic kidney disease | 257 (13.2) | 48 (26.2) | <0.001 | 73 (11.1) | 115 (12.2) | 0.499 |
| Liver disease | 69 (3.5) | 12 (6.6) | 0.042 | 18 (2.7) | 37 (3.9) | 0.199 |
| Heart failure | 145 (7.4) | 16 (8.7) | 0.562 | 68 (10.3) | 107 (11.3) | 0.521 |

Supplementary Table 3. (continued)

| Characteristics | In-hospital Survivor without LST | Non-Survivor without LST | P-value | In-hospital Survivor  with LST | Non-Survivor with LST | P-value |
| --- | --- | --- | --- | --- | --- | --- |
| Chronic obstructive pulmonary  disease | 147 (7.6) | 27 (14.8) | <0.001 | 36 (5.5) | 84 (8.9) | 0.010 |
| Cerebrovascular disease | 513 (26.3) | 41 (22.4) | 0.245 | 198 (30.0) | 218 (23.1) | 0.002 |
| Dementia | 574 (29.5) | 53 (29.0) | 0.883 | 286 (43.4) | 297 (31.5) | <0.001 |
| Suspected infection site of sepsis |  |  |  |  |  |  |
| Pulmonary | 875 (44.9) | 90 (49.2) | 0.271 | 385 (58.4) | 600 (63.6) | 0.038 |
| Abdominal | 468 (24.0) | 33 (18.0) | 0.067 | 100 (15.2) | 182 (19.3) | 0.034 |
| Urinary | 604 (31.0) | 41 (22.4) | 0.015 | 196 (29.7) | 172 (18.2) | <0.001 |
| Skin/soft tissue | 51 (2.6) | 13 (7.1) | <0.001 | 9 (1.4) | 17 (1.8) | 0.497 |
| Catheter-related | 10 (0.5) | 2 (40.0) | 0.317 | 2 (0.3) | 3 (60.0) | 0.960 |
| Systemic infection without  clear primary site of infection | 99 (5.1) | 16 (8.7) | 0.036 | 40 (6.1) | 77 (8.2) | 0.114 |
| Neurologic | 11 (0.6) | 2 (1.1) | 0.381 | 3 (0.5) | 3 (0.3) | 0.657 |
| Type of pathogen |  |  |  |  |  |  |
| Bacteria | 1196 (61.4) | 118 (64.5) | 0.417 | 378 (24) | 536 (56.8) | 0.817 |
| Gram positive bacteria | 358 (18.4) | 48 (26.2) | 0.010 | 112 (17) | 204 (21.6) | 0.022 |

Supplementary Table 3. (continued)

| Characteristics | In-hospital Survivor without LST | Non-Survivor without LST | P-value | In-hospital Survivor  with LST | Non-Survivor with LST | P-value |
| --- | --- | --- | --- | --- | --- | --- |
| Gram negative bacteria | 964 (49.5) | 86 (47) | 0.515 | 312 (47.3) | 400 (42.4) | 0.049 |
| Atypical bacteria | 8 (0.4) | 0 (0.0) | 0.385 | 0 (0.0) | 5 (0.5) | 0.061 |
| Virus | 22 (1.1) | 2 (1.1) | 0.964 | 6 (0.9) | 17 (1.8) | 0.140 |
| Fungus | 63 (3.2) | 19 (10.4) | <0.001 | 11 (1.7) | 28 (3.0) | 0.097 |
| Tuberculosis | 13 (0.7) | 2 (1.1) | 0.511 | 3 (0.5) | 8 (0.8) | 0.349 |
| Others | 6 (0.3) | 1 (0.5) | 0.590 | 1 (0.2) | 0 (0.0) | 0.231 |
| MDR pathogens | 512 (26.3) | 52 (28.4) | 0.535 | 178 (27) | 254 (26.9) | 0.963 |
| Sepsis bundle |  |  |  |  |  |  |
| 1-hour bundle | 289 (14.8) | 33 (18.0) | 0.250 | 115 (17.5) | 156 (16.5) | 0.627 |
| 1-hour Measurement of lactate | 1578 (81.0) | 155 (84.7) | 0.225 | 577 (87.6) | 810 (85.8) | 0.312 |
| 1-hour Blood culture | 1276 (65.5) | 121 (66.1) | 0.874 | 437 (66.3) | 603 (63.9) | 0.315 |
| 1-hour broad spectrum antibiotics | 373 (19.2) | 45 (24.6) | 0.077 | 135 (20.5) | 194 (20.6) | 0.975 |
| 1-hour fluid therapy | 1926 (98.9) | 178 (97.3) | 0.051 | 646 (98) | 917 (97.1) | 0.262 |
| 1-hour use of vasopressor | 1858 (95.4) | 164 (89.6) | <0.001 | 614 (93.2) | 828 (87.7) | <0.001 |
| 3-hour bundle | 1097 (56.3) | 98 (53.6) | 0.467 | 432 (65.6) | 623 (66.0) | 0.854 |
| 3-hour Measurement of lactate | 1782 (91.5) | 169 (92.3) | 0.701 | 632 (95.9) | 897 (95.0) | 0.408 |

Supplementary Table 3. (continued)

| Characteristics | In-hospital Survivor without LST | Non-Survivor without LST | P-value | In-hospital Survivor  with LST | Non-Survivor with LST | P-value |
| --- | --- | --- | --- | --- | --- | --- |
| 3-hour Blood culture | 1716 (88.1) | 163 (89.1) | 0.707 | 588 (89.2) | 837 (88.7) | 0.725 |
| 3-hour broad spectrum antibiotics | 1241 (63.7) | 112 (61.2) | 0.495 | 459 (69.7) | 681 (72.1) | 0.279 |
| 3-hour fluid therapy | 1933 (99.3) | 180 (98.4) | 0.181 | 650 (98.6) | 928 (98.3) | 0.601 |
| 3-hour use of vasopressor | 1904 (97.8) | 177 (96.7) | 0.356 | 632 (95.9) | 889 (94.2) | 0.122 |
| 6-hour bundle | 1581 (81.2) | 140 (76.5) | 0.123 | 583 (88.5) | 814 (86.2) | 0.188 |
| 6-hour Measurement of lactate | 1811 (93.0) | 169 (92.3) | 0.737 | 639 (97.0) | 911 (96.5) | 0.612 |
| 6-hour Blood culture | 1864 (95.7) | 174 (95.1) | 0.677 | 642 (97.4) | 901 (95.4) | 0.040 |
| 6-hour broad spectrum antibiotics | 1745 (89.6) | 157 (85.8) | 0.109 | 612 (92.9) | 874 (92.6) | 0.830 |
| 6-hour fluid therapy | 1938 (99.5) | 180 (98.4) | 0.042 | 650 (98.6) | 930 (98.5) | 0.846 |
| 6-hour use of vasopressor | 1929 (99.1) | 179 (97.8) | 0.107 | 638 (96.8) | 911 (96.5) | 0.736 |
| Appropriateness of initial empiric antibiotics (within 24 hours) | 1709 (87.8) | 160 (87.4) | 0.892 | 566 (85.9) | 766 (81.1) | 0.013 |

Supplementary Table 3. (continued)

| Characteristics | In-hospital Survivor without LST | Non-Survivor without LST | P-value | In-hospital Survivor  with LST | Non-Survivor with LST | P-value |
| --- | --- | --- | --- | --- | --- | --- |
| Admission to the ICU | 722 (37.1) | 105 (57.4) | <0.001 | 172 (26.1) | 415 (44.0) | <0.001 |
| Interventions in the ICU |  |  |  |  |  |  |
| Invasive mechanical ventilation | 238 (12.2) | 72 (39.3) | <0.001 | 65 (9.9) | 270 (28.6) | <0.001 |
| NIV | 34 (1.7) | 5 (2.7) | 0.342 | 6 (0.9) | 15 (1.6) | 0.240 |
| HFNC | 214 (11.0) | 28 (15.3) | 0.079 | 52 (7.9) | 145 (15.4) | <0.001 |
| Continuous renal replacement  therapy | 89 (4.6) | 44 (24.0) | <0.001 | 26 (3.9) | 172 (18.2) | <0.001 |
| ECMO | 2 (0.1) | 1 (0.5) | 0.126 | 0 (0.0) | 0 (0.0) | NA |
| Hemodialysis | 16 (0.8) | 5 (2.7) | 0.012 | 4 (0.6) | 6 (0.6) | 0.943 |

LST; Life sustaining treatment, SOFA; Sequential Organ Failure Assessment, MDR: Multidrug Resistant, ICU; Intensive care unit, NIV; Non-invasive ventilation, HFNC; high flow nasal cannula, ECMO; Extracorporeal membrane oxygenation

Table 4. Univariable and multivariable Cox regression analysis of factors associated with In-hospital mortality – Without LST

|  | Univariate analysis | | | Multivariate analysis | | |
| --- | --- | --- | --- | --- | --- | --- |
|  | HR | 95% CI | P-value | HR | 95% CI | P-value |
| Age, yr | 1.039 | 1.001 – 1.079 | 0.042 | 1.048 | 1.008 – 1.090 | 0.019 |
| Male | 0.771 | 0.564 – 1.054 | 0.103 |  |  |  |
| Body mass index, kg/m^2^ | 0.979 | 0.940 – 1.019 | 0.296 |  |  |  |
| Charlson comorbidity index | 1.138 | 1.080 – 1.200 | <0.001 | 1.040 | 0.969 – 1.117 | 0.275 |
| SOFA score | 1.206 | 1.152 – 1.263 | <0.001 | 1.130 | 1.073 – 1.190 | <0.001 |
| Hematologic malignancy | 2.916 | 1.367 – 6.220 | 0.006 | 3.014 | 1.372 – 6.624 | 0.006 |
| Chronic kidney disease | 2.268 | 1.619 – 3.177 | <0.001 | 1.464 | 0.972 – 2.205 | 0.068 |
| Chronic liver disease | 1.758 | 0.976 – 3.166 | 0.060 | 1.177 | 0.635 – 2.180 | 0.605 |
| Chronic obstructive pulmonary disease | 1.286 | 1.018 – 1.625 | 0.035 | 1.896 | 1.184 – 3.036 | 0.008 |
| Suspected infection site of sepsis |  |  |  |  |  |  |
| Abdominal | 0.805 | 0.547 – 1.186 | 0.272 |  |  |  |
| Urinary | 0.780 | 0.544 – 1.119 | 0.178 |  |  |  |
| Skin/soft tissue | 2.019 | 1.142 – 3.569 | 0.016 | 2.483 | 1.392 – 4.429 | 0.002 |
| Systemic infection without  clear primary site of infection | 1.490 | 0.808 – 2.749 | 0.202 |  |  |  |
|  | Univariate analysis | |  | Multivariate analysis | |  |
|  | HR | 95% CI | P-value | HR | 95% CI | P-value |
| Types of pathogen |  |  |  |  |  |  |
| Gram positive bacteria | 1.290 | 0.914 – 1.821 | 0.148 |  |  |  |
| Fungus | 2.272 | 1.405 – 3.675 | <0.001 | 2.036 | 1.247 – 3.325 | 0.004 |
| 1-hour broad spectrum antibiotics | 1.038 | 0.721 – 1.494 | 0.842 |  |  |  |
| 1-hour fluid therapy | 0.641 | 0.236 – 1.740 | 0.383 |  |  |  |
| 1-hour use of vasopressor | 0.638 | 0.368 – 1.107 | 0.110 |  |  |  |
| 6-hour fluid therapy | 0.576 | 0.140 – 2.365 | 0.444 |  |  |  |
| Admission to the ICU | 1.623 | 1.175 – 2.242 | 0.003 | 0.764 | 0.484 – 1.205 | 0.247 |
| Interventions in the ICU |  |  |  |  |  |  |
| Invasive mechanical ventilation | 2.687 | 1.958 – 3.688 | <0.001 | 2.099 | 1.362 – 3.236 | <0.001 |
| HFNC | 0.963 | 0.639 – 1.451 | 0.856 |  |  |  |
| Continuous renal replacement  therapy | 3.492 | 2.459 – 4.957 | <0.001 | 2.268 | 1.477 – 3.480 | <0.001 |
| Hemodialysis | 2.199 | 0.897 – 5.389 | 0.085 | 0.971 | 0.374 – 2.519 | 0.951 |

LST; Life sustaining treatment, HR; Hazard ratio, CI; Confidence interval, SOFA; Sequential Organ Failure Assessment, ICU; Intensive care unit, HFNC; high flow nasal cannula

Table 5. Univariate and multivariate Cox regression analysis addressing the factors for In-hospital mortality – With LST

|  | Univariate analysis | | | Multivariate analysis | | |
| --- | --- | --- | --- | --- | --- | --- |
|  | HR | 95% CI | P-value | HR | 95% CI | P-value |
| Age, yr | 1.003 | 0.987 – 1.019 | 0.690 |  |  |  |
| Male | 0.951 | 0.828 – 1.093 | 0.478 |  |  |  |
| Resident of nursing care center | 1.003 | 0.839 – 1.198 | 0.978 |  |  |  |
| Charlson comorbidity index | 1.000 | 0.969 – 1.033 | 0.979 |  |  |  |
| Clinical frailty scale | 1.013 | 0.970 – 1.057 | 0.558 |  |  |  |
| SOFA score | 1.066 | 1.042 – 1.091 | <0.001 | 1.061 | 1.036 – 1.086 | <0.001 |
| Solid tumor | 1.137 | 0.972 – 1.330 | 0.109 |  |  |  |
| Chronic obstructive pulmonary disease | 1.286 | 1.018 – 1.625 | 0.035 | 1.237 | 0.976 – 1.567 | 0.078 |
| Cerebrovascular disease | 0.837 | 0.582 – 1.203 | 0.336 |  |  |  |
| Dementia | 0.915 | 0.789 – 1.063 | 0.245 |  |  |  |
| Suspected infection site of sepsis |  |  |  |  |  |  |
| Abdominal | 1.157 | 0.972 – 1.378 | 0.101 |  |  |  |
| Urinary | 0.655 | 0.549 – 0.782 | <0.001 | 0.652 | 0.541 – 0.784 | <0.001 |
| Types of pathogen |  |  |  |  |  |  |
| Gram positive bacteria | 1.159 | 0.982 – 1.368 | 0.080 | 1.067 | 0.901 – 1.263 | 0.454 |
|  | Univariate analysis | |  | Multivariate analysis | |  |
|  | HR | 95% CI | P-value | HR | 95% CI | P-value |
| Gram negative bacteria | 0.851 | 0.739 – 0.979 | 0.024 | 0.934 | 0.801 – 1.088 | 0.379 |
| Atypical bacteria | 2.470 | 1.024 – 5.956 | 0.044 | 2.508 | 1.036 – 6.069 | 0.041 |
| Fungus | 1.582 | 1.077 – 2.323 | 0.019 | 1.363 | 0.923 – 2.013 | 0.119 |
| 1-hour use of vasopressor | 0.631 | 0.501 – 0.794 | <0.001 | 0.652 | 0.517 – 0.822 | <0.001 |
| Appropriateness of initial empiric therapy (within 24 hours) | 0.858 | 0.720 – 1.022 | 0.086 | 0.799 | 0.663 – 0.962 | 0.018 |
| Admission to the ICU | 1.126 | 0.980 – 1.294 | 0.095 | 0.772 | 0.631 – 0.945 | 0.012 |
| Invasive mechanical ventilation | 1.385 | 1.196 – 1.604 | <0.001 | 1.288 | 1.047 – 1.585 | 0.017 |
| HFNC | 0.991 | 0.828 – 1.187 | 0.926 |  |  |  |
| Continuous renal replacement  therapy | 1.513 | 1.277 – 1.792 | <0.001 | 1.458 | 1.192 – 1.785 | 0.001 |

LST; Life sustaining treatment, HR; Hazard ratio, CI; Confidence interval, SOFA; Sequential Organ Failure Assessment, ICU; Intensive care unit, HFNC; high flow nasal cannula
